# Idiopathic Intracranial Hypertension With Papilledema and Iron Deficiency Anemia in a 14-Year-Old Female Patient: A Case Report of First Presentation in the Middle East

**DOI:** 10.7759/cureus.82346

**Published:** 2025-04-16

**Authors:** Gunjan Awatramani, Ruzaina Sait, Ghazal Talal Saeed, Sara Vidha, Zainab Al-Abdullah, Ghazaleh Ghaffaripour, Mahmoud Marashi, Pramod Warhekar

**Affiliations:** 1 College of Medicine, Mohammed Bin Rashid University of Medicine and Health Sciences, Dubai, ARE; 2 Neuroscience Research Center, Iran University of Medical Sciences, Tehran, IRN; 3 Hematology, Mediclinic City Hospital, Dubai, ARE; 4 Ophthalmology, Mediclinic City Hospital, Dubai, ARE

**Keywords:** anemia, benign intracranial hypertension, general ophthalmology, idiopathic intracranial hypertension (iih), iron deficiency anemia (ida), ophthalmology journal, pediatric neurology, pediatric ophthalmology & strabismus

## Abstract

Idiopathic intracranial hypertension (IIH) in the setting of iron deficiency anemia (IDA) is a rare association, possibly attributed to changes in cerebrospinal fluid (CSF) dynamics due to either iron homeostasis or blood hyperviscosity, although a definitive causal relationship continues to be speculated upon. This case report presents a rare instance of IDA causing IIH with accompanying papilledema in a 14-year-old female patient, who is a known case of polycystic ovarian syndrome. The patient presented to an outpatient clinic with tension-like headaches. Neurological examination, including fundoscopy, was significant for bilateral papilledema. Investigations revealed IDA. The patient was managed with acetazolamide and iron supplementation for her concurrent anemia. Despite initial management, her headaches persisted, and her papilledema progressed. Due to this progression, lumbar puncture was performed and confirmed elevated intracranial pressure (ICP). After completing an adjusted regimen of acetazolamide and iron supplementation, both the patient's symptoms and papilledema resolved and did not recur. This report encourages further research to support the potential connection of IDA with IIH and aid clinicians in promptly diagnosing and managing similar cases.

## Introduction

Idiopathic intracranial hypertension (IIH), also known as pseudotumor cerebri, is a diagnosis of exclusion in the workup of increased intracranial pressure (ICP). The incidence of IIH has increased greatly from 2.3/100,000/year in 2003 to 7.8/100,000/year in 2017 [1]. Associated risk factors include obesity, women of childbearing age, endocrine conditions, which include Addison’s disease and hyperparathyroidism, as well as the use of hormonal contraceptives, to name a few [2]. Symptoms typically include headaches, which are possibly associated with nausea and vomiting, diplopia, transient vision loss, pulsatile tinnitus, and permanent vision loss in severe cases [3]. The diagnosis of IIH is based on several factors, including normal cerebrospinal fluid (CSF) composition, increased CSF opening pressure greater than 250 mm upon lumbar puncture, and an unremarkable neurological examination. On ophthalmic examination, fundoscopy typically reveals optic disc edema, while visual acuity is used to assess the impact on the vision, and the perimetry helps to identify enlarged blind spots present in this condition. Magnetic resonance imaging is used to rule out secondary causes of increased ICP [3]. Management is most commonly pharmacological, using carbonic anhydrase inhibitors such as acetazolamide [4,5].

Papilledema is defined as optic disc swelling due to increased ICP, typically present bilaterally and symmetrically on fundoscopy [6]. The diagnosis is made using the modified Dandy’s criteria with the Frisén classification for papilledema staging [7]. Etiologies of papilledema include IIH, intracranial lesions, cerebral venous sinus thrombosis, meningitis, hydrocephalus, malignant hypertension, medication-induced papilledema, along with other causes of increased ICP [8]. Management involves treating the underlying cause of the increased ICP [9].

Iron deficiency anemia (IDA) refers to a low blood hemoglobin concentration or red blood cell count attributed to reduced iron reserves in the human body. The normal range of blood iron concentration is 10-30 umol/L. IDA is defined as a microcytic, hypochromic anemia, mainly due to any etiology causing blood loss (such as menstruation), iron absorption pathologies (i.e., celiac disease, tropical sprue, inflammatory bowel disease), reduced dietary iron intake, and conditions leading to increased iron demand (i.e., pregnancy, breastfeeding, menstruation) [10].

The relationship of IDA with IIH and/or papilledema is speculative due to limited evidence from existing literature supporting it. Proposed explanations from the literature suggest that anemia results in a hyperviscous state with increased venous pressure, therefore resulting in increased ICP [9]. We present a rare case of IIH with accompanying papilledema and a history of IDA.

## Case presentation

A 14-year-old female patient with a two-year history of menorrhagia and polycystic ovarian syndrome (PCOS) presented to an outpatient clinic with a five-day history of moderately intense headaches described as pressure-like, originating from the left frontal area and radiating to the back of the ear. The pain would start in the middle of the day, last an hour, and then recur in the evening with similar characteristics. No associated symptoms such as photophobia, phonophobia, ophthalmalgia, and nausea or vomiting were reported. She reported no changes in vision, smell, gait, or balance.

With regard to her menstrual history, she attained menarche at the age of 12, experienced irregular cycles initially, and continued to have heavy menses and irregular cycles beyond one year after attaining menarche. Family history was significant for IDA in her mother. Conditions that contribute to IDA, such as Crohn's disease and celiac disease, were ruled out through a comprehensive history of the patient's symptoms. Her diet was healthy and varied.

**Figure 1:**
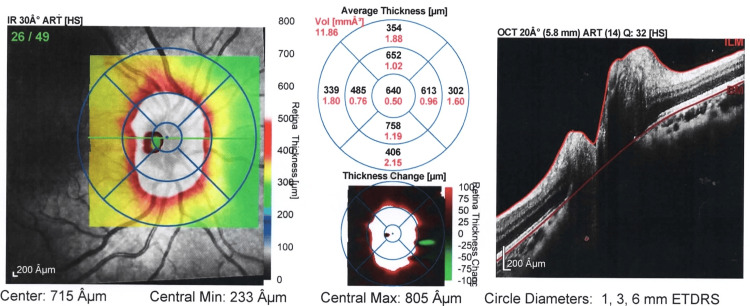
Image representing thickness map report OD at presentation of patient. The average central optic disc thickness is 300-400 µm in healthy eyes. In this patient, the average central optic disc thickness is 640 µm suggesting severe swelling of the optic disc OD: right eye

**Figure 2:**
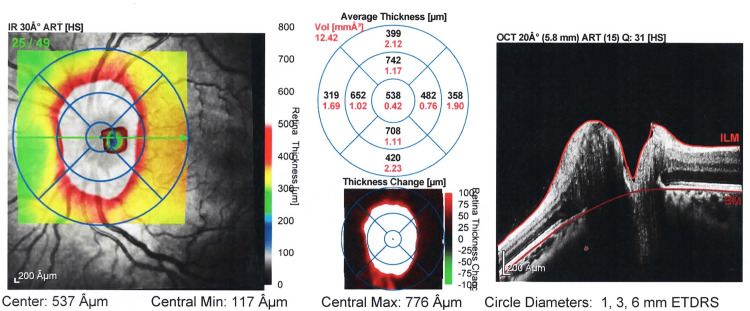
Image representing thickness map report OS at presentation of patient. In this image, the average central optic disc thickness is 538 µm suggesting severe swelling of the optic disc bilaterally OS: left eye

Vital signs during initial presentation were within normal limits. Her weight was 61.4 kg, and height was 158.5 cm, making her BMI 24.4 kg/m², placing her in the 86th percentile for her age, which classifies her as overweight. Neurological examination, including cranial nerve examination, was unremarkable. On ophthalmological examination, ocular movements were full in all directions with orthophoria. Pupils were equal and reactive to light, with no intranuclear ophthalmoplegia noted. Fundoscopic examination was significant for bilateral papilledema of Grade 3 (moderate severity), predominantly in the nasal region. The optic cup-to-disc ratio was observed to be > 0.53 (normal less than 0.5). There were no other significant findings, such as fluffy exudates or hemorrhages on fundoscopy. An optical coherence tomography (OCT) was performed when she initially presented to the clinic, which demonstrated elevated central optic disc height bilaterally, as demonstrated in Figure 1 and Figure 2. Visual field tests were unremarkable.

She was then referred to a pediatric neurologist who recommended proceeding with an MRI brain with contrast and a visual evoked potential (VEP) test. MRI brain with contrast reported no signs of obstructive hydrocephalus or intracerebral masses, and VEP results were reported as unremarkable. Cerebral magnetic resonance angiogram and venogram were also performed, which helped rule out the possible presence of thrombosis. Lumbar puncture could not be performed initially due to a lack of parental consent.

With regard to laboratory investigations, the hemoglobin level was noted to be 9.1 g/dL (12-16 g/dL), MCV was 76.3 fl (80-100 fl), and a ferritin of <1.0 ng/mL (20.00- 120.00 ng/mL), which confirmed a diagnosis of IDA. Peripheral blood smear showed microcytic hypochromic RBCs with mild anisopoikilocytosis (Table 1).

**Table 1:** Lab investigations with results at presentation of the patient HCT/PCV: hematocrit/packed cell volume; RBC: red blood cell; MCH: mean corpuscular hemoglobin; MCHC: mean corpuscular hemoglobin concentration; MCV: mean corpuscular volume

Test	Patient Results	Normal Ranges
Hemoglobin	9.1 g/dL	12-16 g/dL
HCT/PCV	29.4 %	36.00-46.00 %
RBC	3.85 M/ul	4.10-5.10 M/ul
Ferritin	<1.0 ng/mL	20.00-120.00 ng/mL
MCH	23.7 pg	25.0-35.0 pg
MCHC	31.0 g/dL	31.0-37.0 g/dL
MCV	76.3 fl	78.0-100.0 fl

A provisional diagnosis of IIH was made. The patient was prescribed a course of once-daily 250 mg acetazolamide and 600 mg potassium chloride tablets once daily to reduce the ICP. For management of her IDA, she was prescribed intravenous iron supplementation and was advised to increase consumption of fruits rich in potassium for two weeks. 

Despite this, the patient continued to complain of mild headache and dizziness, and her bilateral papilledema was observed to have progressed in the subsequent follow-up visit two weeks later. Lumbar puncture was then performed at this stage with an elevated opening pressure of 38 cm H₂O (10-20 cm H₂O) and a closing pressure of 10 cm H₂O (6-25 cm H₂O). CSF analysis yielded normal results.

The dose of acetazolamide was increased to 500 mg three times daily, and paracetamol was added as needed to her regimen to manage headaches. Repeat OCT and field tests in the days post-lumbar puncture showed no signs of worsening.

**Figure 3:**
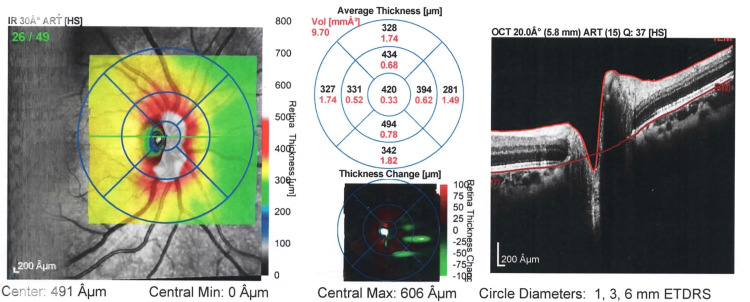
Image representing thickness map report OD following treatment of patient with iron deficiency anemia OD: right eye

**Figure 4:**
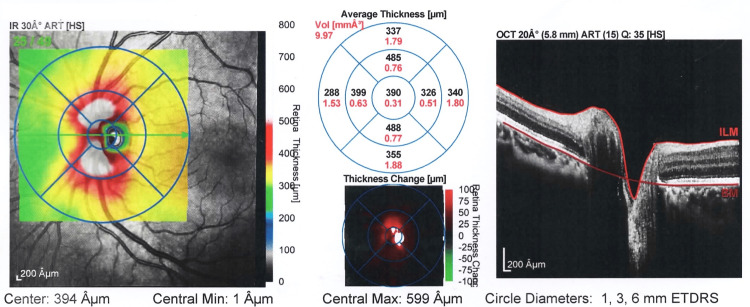
Image representing thickness map report OS following treatment of patient with iron deficiency anemia OS: left eye

After two weeks, the patient’s condition improved vastly with the modified prescription and the lumbar puncture. Visual fields remained stable. Disc OCT showed improvement in papilledema bilaterally, and hence, acetazolamide was slowly tapered down over four months. Disc OCT performed after the fifth month showed a stable disc OCT elevation bilaterally, as evidenced in Figure 3 and Figure 4, and therefore, her medication regimen of acetazolamide and potassium chloride was stopped indefinitely. Subsequently, with regular iron injections, her anemia improved, and she was switched to oral iron supplements for maintenance. In her most recent visit, which was two years after her initial presentation, the patient is doing well with complete resolution of the bilateral papilledema.

## Discussion

IIH is signified by the presence of elevated ICP, which thereby leads to the symptoms of headache, pulsatile tinnitus, as well as vision loss and diplopia [11]. Risk factors of IIH include females of childbearing age with an elevated BMI, which was present in our patient [12]. The diagnosis of IIH is made with a normal neurological examination, normal CSF composition, increased opening pressure on lumbar puncture, supplemented with normal brain parenchyma on imaging, and the possible presence of papilledema [13]. This case report demonstrates the presence of papilledema in a female patient suffering from IDA caused by heavy menses. It will also be the first of its kind to be reported within the Middle East region and will add to the growing literature of such patient presentations.

The causal relationship between IDA and IIH remains undetermined. However, multiple case reports have been reported globally, demonstrating the presence of papilledema alongside IDA. One such case was reported in the United Kingdom, where a 31-year-old female patient, initially presenting with anemia symptoms, also developed symptoms of raised ICP and was diagnosed with papilledema [14]. In Canada, five female patients were reported to have IIH secondary to new-onset IDA. Out of these patients, two were not found to be obese. Prompt resolution of the papilledema in these cases occurred following treatment of their anemia. Of note, there was no change in weight over the course of their treatment, suggesting that anemia played a role in the presence of IIH [15]. Similarly, the patient in this case report had improvement of papilledema bilaterally following treatment for her IDA.

Various pathophysiological mechanisms have been suggested regarding the possible relationship between IIH and IDA, which include a potential hyperviscous state leading to increased cerebral venous pressure and therefore increased ICP [16]. Another suggested mechanism involves alterations in CSF secondary to dysfunctional iron states in the body and hence a raised ICP [17]. The third suggested mechanism is the presence of tissue hypoxia, causing raised ICP due to increased brain capillary permeability [18].

As reported by Mollan et al. in an article where 77 cases of IIH were identified, eight cases were found to have microcytic anemia with raised ICP. The outcome of one patient following treatment was poor, with progressive visual loss, requiring a ventriculoperitoneal shunt [17]. In most cases of IIH associated with anemia, prompt correction of the anemia itself can result in resolution of the papilledema, with rare progression requiring surgical intervention. In a study by Ma et al., most patients treated for anemia showed correction of their vision, papilledema, and CSF opening pressure [19].

Specifically, the presence of papilledema in pediatric patients secondary to IDA has not been extensively reported in the literature. However, case reports of papilledema in the presence of other types of anemia, such as autoimmune hemolytic anemia and aplastic anemia, have been reported with similarly described pathophysiological mechanisms [20]. The long-term sequelae and management outcomes have not been extensively delved into.

## Conclusions

This case report, the first of its kind documented in the Middle East, describes a patient who experienced a possible exacerbation of IIH with papilledema due to IDA. She underwent multidisciplinary management, which included adding iron supplementation to treat the IDA, acetazolamide, and potassium chloride, along with therapeutic and diagnostic lumbar puncture to manage symptoms caused by raised ICP. Our case report does not provide evidence that there is a definite causal relationship between IIH with papilledema and IDA. However, there is an association between the presence of anemia and papilledema that needs to be delved into. This case report aims to encourage further research surrounding the connection and aid clinicians encountering similar cases.
